# Association of Religious and Spiritual Factors With Patient-Reported Outcomes of Anxiety, Depressive Symptoms, Fatigue, and Pain Interference Among Adolescents and Young Adults With Cancer

**DOI:** 10.1001/jamanetworkopen.2020.6696

**Published:** 2020-06-16

**Authors:** Daniel H. Grossoehme, Sarah Friebert, Justin N. Baker, Matthew Tweddle, Jennifer Needle, Jody Chrastek, Jessica Thompkins, Jichuan Wang, Yao I. Cheng, Maureen E. Lyon

**Affiliations:** 1 Rebecca D. Considine Research Institute, Akron Children’s Hospital, Akron, Ohio; 2 Haslinger Family Pediatric Palliative Care Center, Akron Children’s Hospital, Akron, Ohio; 3 Division of Quality of Life and Palliative Care, Department of Oncology, St Jude Children’s Research Hospital, Memphis, Tennessee; 4 Chaplaincy Services, Akron Children’s Hospital, Akron, Ohio; 5 Division of Pediatric Critical Care Medicine, University of Minnesota Health, Minneapolis; 6 Pediatric Palliative Care, University of Minnesota Health–Fairview, Minneapolis; 7 Division of Adolescent and Young Adult Medicine, Children’s National Hospital, Washington, DC; 8 Center for Translational Research at Children’s National Research Institute, Washington, DC; 9 Division of Biostatistics and Study Methodology, Center for Translational Research at Children’s National Research Institute, Washington, DC; 10 George Washington University School of Medicine and Health Sciences, Washington, DC

## Abstract

**Importance:**

The associations of spiritual and religious factors with patient-reported outcomes among adolescents with cancer are unknown.

**Objective:**

To model the association of spiritual and religious constructs with patient-reported outcomes of anxiety, depressive symptoms, fatigue, and pain interference.

**Design, Setting, and Participants:**

This cross-sectional study used baseline data, collected from 2016 to 2019, from an ongoing 5-year randomized clinical trial being conducted at 4 tertiary-referral pediatric medical centers in the US. A total of 366 adolescents were eligible for the clinical trial, and 126 were randomized; participants had to be aged 14 to 21 years at enrollment and be diagnosed with any form of cancer. Exclusion criteria included developmental delay, scoring greater than 26 on the Beck Depression Inventory II, non-English speaking, or unaware of cancer diagnosis.

**Exposures:**

Spiritual experiences, values, and beliefs; religious practices; and overall self-ranking of spirituality’s importance.

**Main Outcomes and Measures:**

Variables were taken from the Brief Multidimensional Measurement of Religiousness/Spirituality (ie, feeling God’s presence, daily prayer, religious service attendance, being very religious, and being very spiritual) and the spiritual well-being subscales of the Functional Assessment of Chronic Illness Therapy (meaning/peace and faith). Predefined outcome variables were anxiety, depressive symptoms, fatigue, and pain interference from Patient-Reported Outcomes Measurement Information System pediatric measures.

**Results:**

A total of 126 individuals participated (72 [57.1%] female participants; 100 [79.4%] white participants; mean [SD] age, 16.9 [1.9] years). Structural equation modeling showed that meaning and peace were inversely associated with anxiety (β = –7.94; 95% CI, –12.88 to –4.12), depressive symptoms (β = –10.49; 95% CI, –15.92 to –6.50), and fatigue (β = –8.90; 95% CI, –15.34 to –3.61). Feeling God’s presence daily was indirectly associated with anxiety (β = –3.37; 95% CI, –6.82 to –0.95), depressive symptoms (β = –4.50; 95% CI, –8.51 to –1.40), and fatigue (β = –3.73; 95% CI, –8.03 to –0.90) through meaning and peace. Considering oneself very religious was indirectly associated with anxiety (β = –2.81; 95% CI, –6.06 to –0.45), depressive symptoms (β = −3.787; 95% CI, –7.68 to –0.61), and fatigue (β = –3.11, 95% CI, –7.31 to –0.40) through meaning and peace. Considering oneself very spiritual was indirectly associated with anxiety (β = 2.11; 95% CI, 0.05 to 4.95) and depression (β = 2.8, 95% CI, 0.07 to 6.29) through meaning and peace. No associations were found between spiritual scales and pain interference.

**Conclusions and Relevance:**

In this study, multiple facets of spirituality and religiousness were associated with anxiety, depression, and fatigue, all of which were indirectly associated with the participant’s sense of meaning and peace, which is a modifiable process. Although these results do not establish a causal direction, they do suggest palliative interventions addressing meaning-making, possibly including a spiritual or religious dimension, as a novel focus for intervention development.

## Key Points

**Question:**

Among adolescents and young adults with cancer, is there an association between spirituality and patient-reported outcomes, and are these outcomes associated with a sense of meaning, peace, and comfort provided by faith?

**Findings:**

In this cross-sectional study of 126 adolescents and young adults with cancer, structural equation modeling revealed that meaning and peace were associated with aspects of spirituality and religiousness as well as anxiety, depressive, and fatigue symptoms.

**Meaning:**

In this study, participants’ sense of meaning and peace was associated with religiousness and with anxiety and depression, possibly representing an underappreciated intervention target.

## Teaser

This cross-sectional study models the association of spiritual and religious constructs with patient-reported outcomes of anxiety, depressive symptoms, fatigue, and pain interference.

## Introduction

Cancer is the leading cause of disease-related death among US residents aged 15 to 24 years.^1^ Despite optimism regarding improving remission rates, adolescents and young adults in treatment continue to experience numerous adverse effects as well as physical, psychological, social, and spiritual comorbidities.^2^ Adolescents report anxiety, depressive symptoms, fatigue, and pain interference as among their most debilitating patient-reported outcomes (PROs).^3,4,5^ Palliative care provides symptom management and psychosocial support for patients with serious illness and their families. Symptom management is person-centered and therefore spans the biopsychosocial-spiritual spectrum of concerns.^6^

Coping with anxiety, depressive symptoms, fatigue, and pain interference is multifaceted. Approaches to palliation may be pharmaceutic, psychologic, and, for many people, spiritual. Most of the US population (75%) reports spirituality is at least somewhat important, and 53% report that it is very important to them.^7^ Many adolescents and young adults (AYAs) report shifting from institutional religion toward a broader spirituality^8^; this does not mean these issues are unimportant. In a qualitative study of 17 AYAs (aged 14 to 25 years) with cancer,^9^ only 31% reported that they were neither spiritual nor religious; most identified as spiritual (whether religious or not).

Spirituality has shown mixed associations with health outcomes. Spiritual struggles^10^ have almost consistently been associated with poorer health outcomes, especially mental health outcomes, in both AYAs and adults.^11,12,13,14,15^ Studies of religion, spirituality, and anxiety in AYAs are few and have shown mixed results depending on how religion and spirituality were operationalized and whether state vs trait anxiety was measured.^16^ A Canadian study showed AYAs aged 15 years and older with pain who were both spiritual and religious had better psychologic well-being scores and used positive coping methods more than those who were not.^17^

Religion and spirituality are recognized as integral components of palliative care practice, although they may be underappreciated constructs in other fields.^6^ Religion and spirituality are multidimensional and commonly studied in bivariate analyses with health outcomes. What is unknown are the potential pathways by which religious and spiritual constructs affect PROs. This hinders designing, developing, prototyping, and testing palliative interventions that include religion and spirituality to improve PROs among AYAs with cancer. When cancer occurs during adolescence or young adulthood, there are long-term consequences to adjustment, functioning, and disease self-management. It is important to understand the potential role religion and/or spirituality play in 4 outcomes AYAs with cancer indicate are the most debilitating (ie, anxiety, depression, pain interference, and fatigue) to provide comprehensive therapies. The specific aims of this study were to examine how religious and spiritual factors were individually associated with anxiety, depression, pain interference, and fatigue PROs and to develop a model of how factors comprising spiritual well-being might lie in the pathway of the association of religious and spiritual factors with anxiety, depression, pain interference, and fatigue among AYAs with cancer.

## Methods

### Participants

This cross-sectional study used existing data from participants at baseline in a 4-site, prospective, longitudinal randomized clinical trial of the Family Centered Advance Care Planning for Teens with Cancer intervention.^18^ The sample size for the primary study was determined by a power analysis for the primary outcomes. Data were collected at enrollment, occurring between July 16, 2016, and April 30, 2019. The sites were pediatric tertiary referral hospitals (Children’s National Hospital, Washington, DC; Akron Children’s Hospital, Akron, Ohio; St Jude Research Hospital, Memphis, Tennessee; and the University of Minnesota Health, Minneapolis). The primary trial was approved by the institutional review boards at participating institutions. Participants were a convenience sample of AYAs with any type of cancer who were treated at these sites at any point after their diagnosis. Inclusion criteria were that participants be aged 14 to 21 years at enrollment; not known to have any developmental delay; have a Beck Depression Inventory total score less than 26; have English as their primary language; not be actively suicidal, homicidal, or psychotic; and be aware of their cancer diagnosis.

### Procedures

#### Methods

After obtaining informed consent from a family member of participants aged 14 to 17 years and assent from the participants and the consent of AYAs aged 18 years and older, baseline data were collected from participants at their first visit. With the AYA alone in a private room, research staff read the questions aloud to the AYA and entered the response into REDCap, unless AYAs requested to enter their own responses.^19^ This approach controls for issues of literacy, health literacy, and uncorrected vision, while serving as an engagement strategy and maximizing likelihood of obtaining complete data.

#### Measures

Covariates (and potential confounders) obtained by medical record review or self-report (ie, age, sex, race, ethnicity, time since diagnosis, treatment status [ie, completed or ongoing], education, household income, and study site) and participants’ self-ranking of the importance of religion and spirituality to them were collected. We collected 3 additional measures, as follows: the Patient-Reported Outcomes Measurement Information System (PROMIS),^20,21,22^ the spiritual well-being scale of the Functional Assessment of Chronic Illness Therapy–Version 4 (FACIT-Sp),^23^ and the Brief Multidimensional Measurement of Religiousness/Spirituality (BMMRS).^24,25^

##### PROMIS

We used 4 of the short-form, pediatric PROMIS symptom measures (ie, emotional distress–anxiety; emotional distress–depressive symptoms; fatigue; and pain interference), which were developed and validated for use with pediatric patients to assess health-related quality of life, as the outcome measures in the study. The psychometric properties of these measures when used with pediatric patients with cancer are established.^20,21,26^ The measures were developed to yield scores on a T-score scale with a mean of 50 and SD of 10.

##### FACIT-Sp

This 23-item nontheistic scale is frequently used to study spirituality and has good psychometric properties among adolescents with chronic illness.^27,28^ The FACIT-Sp has 2 subscales: meaning and peace (items 1-8; eg, “I feel peaceful,” “I have a reason for living”) and faith (items 9-12; eg, “I find comfort/strength in my faith,” “My illness has strengthened my faith or spiritual beliefs”). A higher score indicates better spiritual well-being.

##### BMMRS

The BMMRS is a multidimensional, 38-item, self-administered questionnaire evaluating religious and spiritual dimensions. The scale has demonstrated validity and reliability among adolescent populatons.^15,25,27^ For the purposes of this study, investigators a priori identified 5 items associated with and sensitive to quality of life outcomes^29,30^ and end-of-life decision-making^31^ based on their previous research with adolescents,^30^ as follows: feeling God’s presence; praying privately; attending religious services; identifying as religious; and identifying as spiritual. A higher score indicates higher religiousness and/or spirituality.

### Statistical Analysis

Descriptive sample statistics were reported for social demographic characteristics and outcomes. The religious and spiritual measures were dichotomized (Table 1). The binary associations of religious and spiritual measures with patient self-reported PROMIS measures (ie, anxiety, depression, pain interference, and fatigue) were explored using *t* tests. Structural equation modeling was used to estimate the conceptual model (specified a priori and depicted in the eFigure in the Supplement), in which religious and spiritual measures were associated with the PROMIS symptoms through 2 latent variables (meaning and peace with 8 indicators and faith with 4 indicators, as shown in Table 2), respectively. The basis for the model specification was prior work positing meaning and peace between religious and spiritual beliefs and practices and health outcomes, and work on meaning and peace and depression as well as spiritual struggle and depresson.^32,33,34,35^

**Table 1.** Selected Results of Structural Equation Modeling Using Bayesian Approach^a^

Variable	β (95% CI)										
	PROMIS anxiety			PROMIS depression			PROMIS pain interference			PROMIS fatigue	
	Association through faith		Association through meaning and peace	Association through faith	Association through meaning and peace		Association through faith		Association through meaning and peace	Association through faith	Association through meaning and peace
Association with faith	0.94 (–1.21 to 3.31)		NA	0.84 (–1.30 to 3.31)	NA		0.67 (–1.88 to 3.40)		NA	2.12 (–0.85 to 5.46)	NA
Association with meaning and peace	NA		–7.94 (–12.88 to –4.12)^b^	NA	–10.49 (–15.92 to –6.50)^b^		NA		–2.47 (–7.20 to 2.06)	NA	–8.901 (–15.34 to –3.61)^b^
Feel God’s presence	0.82 (–1.17 to 3.26)	–3.37 (–6.82 to –0.95)^b^		0.74 (–1.23 to 3.22)		–4.50 (–8.51 to –1.40)^b^	0.59 (–1.80 to 3.38)	–0.96 (–3.60 to 0.87)		1.88 (–0.77 to 5.56)	–3.73 (–8.03 to –0.90)^b^
Daily prayer	0.05 (0.55 to 0.98)	0.42 (–1.86 to 2.88)		0.04 (–0.54 to 0.92)		0.58 (–2.43 to 3.69)	0.03 (–0.61 to 0.98)	0.06 (–0.79 to 1.35)		0.15 (–0.86 to 1.75)	0.45 (–2.12 to 3.35)
Religious service	–0.7 (–1.20 to 0.46)	0.81 (–1.44 to 3.29)		–0.05 (–1.02 to 0.49)		1.10 (–1.87 to 4.29)	–0.03 (–1.02 to 0.67)	0.15 (–0.61 to 1.64)		–0.19 (–1.88 to 0.80)	0.87 (–1.61 to 3.91)
Very religious	0.66 (–0.95 to 2.81)	–2.81 (–6.06 to –0.45)^b^		0.58 (–1.07 to 2.67)		–3.77 (–7.68 to –0.61)^b^	0.46 (–1.61 to 2.75)	–0.78 (–3.22 to 0.73)		1.50 (–0.67 to 4.61)	–3.11 (–7.31 to –0.40)^b^
Very spiritual	0.10 (–0.39 to 1.20)	2.11 (0.05 to 4.95)^b^		0.09 (–0.41 to 1.15)		2.84 (0.07 to 6.29)^b^	0.05 (–0.59 to 1.10)	0.56 (–0.58 to 2.49)		0.27 (–0.60 to 1.99)	2.33 (0.04 to 5.82)^b^

Abbreviations: NA, not applicable; PROMIS, Patient Reported Outcomes Measurement System.

^a^ After 50 000 iterations, the highest potential scale reduction was 1.008. 95% CI for the difference between the observed and the replicated χ^2^ values, -46.547, 64.334; posterior predictive *P* = .37.

^b^ *P* < .05.

**Table 2.** Descriptive Statistics of Variables Used in Model Among 126 Adolescents and Young Adults

Variable	Statistics
PROMIS measures, mean (SD)	
PROMIS anxiety T-score	46.7 (9.4)
PROMIS depression T-score	45.0 (9.9)
PROMIS pain interference T-score	43.8 (10.3)
PROMIS fatigue T-score	45.4 (12.5)
FACIT-Sp measures, mean (SD)	
Meaning and peace: 8 items; Cronbach α = 0.79	
1. I feel peaceful	3.1 (1.0)
2. I have a reason for living	3.8 (0.5)
3. My life has been productive	3.3 (0.9)
4. I have trouble feeling peace of mind^a^	3.2 (1.2)
5. I feel a sense of purpose in my life	3.6 (0.8)
6. I am able to reach down deep into myself for comfort	3.1 (1.0)
7. I feel a sense of harmony within myself	2.9 (1.0)
8. My life lacks meaning and purpose^a^	3.6 (0.9)
Faith: 4 items; Cronbach α = 0.79	
9. I find comfort in my faith or spiritual beliefs	2.9 (1.3)
10. I find strength in my faith or spiritual beliefs	2.8 (1.4)
11. My illness has strengthened my faith or spiritual beliefs	2.6 (1.6)
12. I know that whatever happens with my illness, things will be okay	3.5 (0.8)
Brief MMRS variable, No. (%)	
1. I feel God's presence	
Many times a day or every day	39 (31.0)
Most days, some days, once in a while, or never or almost never	83 (65.8)
12. How often do you pray privately; that is, how often do you pray in settings other than a church, synagogue, mosque, or other place of worship and at times when you are not attending functions of a religiously based group?	
More than once a day or once a day	37 (29.4)
A few times a week, once a week, a few times a month, once a month, less than once a month, or never	87 (69.1)
34. How often do you go to religious services?	
More than once a week or every week or more often	41 (32.5)
Once or twice a month, every month or so, once or twice a year, or never	82 (65.1)
37. To what extent do you consider yourself a religious person?	
Very religious or moderately religious	72 (57.1)
Slightly religious or not religious at all	54 (42.9)
38. To what extent do you consider yourself a spiritual person?	
Very spiritual or moderately spiritual	68 (53.9)
Slightly spiritual or not spiritual at all	58 (46.0)

Abbreviations: FACIT-Sp, spiritual well-being subscales of the Functional Assessment of Chronic Illness Therapy; MMRS, Multidimensional Measurement of Religiousness/Spirituality; PROMIS, Patient-Reported Outcomes Measurement Information System.

^a^ Scores reversed, so that 0 indicates very much and 4 indicates not at all.

The structural equation model was estimated using Bayesian estimator in Mplus 8.3 (Muthén and Muthén) that has superior performance in small samples without reliance on asymptotic and data normality assumptions.^36,37,38,39^ The Bayesian approach is a full-information estimator using all available data under a missing-at-random assumption. Such a full information approach is superior to traditional approaches, such as listwise deletion, pairwise deletion, and similar response pattern imputation.^40,41,42^ The missing-at-random assumption is more plausible than the missing-completely-at-random assumption in traditional statistical methods, allowing missingness to be associated with observed covariates and/or outcome measures.^37,39,43^

The convergence of Bayesian estimation was evaluated by potential scale reduction (PSR). If PSR is close to 1 (eg, between 1.0 and 1.1) for all parameters in the model, it indicates that convergence has been achieved. The goodness of fit of the model was assessed by posterior predictive checking.^44^ If the model fits data well, the 95% CI of the difference between the observed and replicated χ^2^ statistics should center around 0, and the posterior predictive *P* value should be greater than .05.^38,44^ Statistical inferences were made by examining the range of parameter estimates that captured 95% of the posterior probability distribution (ie, 95% Bayesian credibility interval, CrI). If the 95% CrI of a parameter estimate did not cover 0, then it was considered statistically significant at 2-tailed α = .05 level.^39,45,46^

## Results

A total of 336 AYAs with cancer who were potentially eligible for the parent study were approached. Of these, 203 AYA (60.4%) declined and 3 (0.9%) did not meet secondary eligibility criteria for decision-making, yielding an enrollment rate of 38.7%. Eligible individuals who declined to indicate their racial identification also declined participation in the trial. A total of 126 AYAs completed baseline assessments; the sample had a mean (SD) age of 16.9 (1.9) years, with 72 (57.1%) female participants, 100 (79.4%) white participants, and 76 (60.3%) Protestant participants. Demographic characteristics are presented in Table 2. Descriptive statistics for PROMIS, FACIT-Sp, and religious and spiritual measures are provided in Table 3; binary associations between PROMIS and religious and spiritual measures appear in Table 1. The internal consistency of the FACIT-Sp subscale measures was good, with a Cronbach α of 0.79 for meaning and peace as well as faith. Binary associations of PROMIS with religious and spiritual measures are reported in Table 1.

**Table 3.** Demographic and Medical Data for Individuals Enrolled and Eligible but Declining to Participate in the Study

Characteristic	No (%)		*P* value
	Enrolled (n = 126)	Declining to participate (n = 203)^a^	
Age, mean (SD), y	16.9 (1.9)	16.7 (1.9)	.21^b^
Religious affiliation			
Agnostic/atheist/none	24 (19.0)	NA	NA
Christian	90 (71.4)	NA	NA
Hindu	1 (0.8)	NA	NA
Jehovah’s Witness	1 (0.8)	NA	NA
Jewish	1 (0.8)	NA	NA
LDS/Mormon	6 (4.8)	NA	NA
Missing	3 (2.4)	NA	NA
Sex			
Female patients	72 (57.1)	82 (41.4)	.01^c^
Male patients	54 (42.9)	115 (58.1)	
Declined to answer	0	1 (0.5)	
Race			
American Indian or Alaska Native	0	1 (0.5)	.23^c^
Asian	3 (2.4)	4 (2.1)	
Black or African American	17 (13.5)	20 (10.4)	
White	100 (79.4)	158 (82.3)	
>1 race	5 (4.0)	2 (1.0)	
Declined to answer	1 (0.8)	7 (3.6)	
Ethnicity			
Hispanic or Latino	5 (4.0)	6 (3.1)	.88^d^
Not Hispanic or Latino	116 (92.1)	176 (92.1)	
Declined to answer	5 (4.0)	9 (4.7)	
Diagnosis			
Leukemia	42 (33.3)	51 (26.7)	.41^c^
Lymphoma	19 (15.1)	39 (20.4)	
Solid tumors	34 (27.0)	47 (24.6)	
Brain tumor	25 (19.8)	38 (19.9)	
Other	6 (4.8)	12 (6.3)	
Unknown	0	4 (2.1)	
On active treatment^e^			
Yes	27 (21.4)	12 (17.6)	.53^d^
No	99 (78.6)	56 (82.4)	

Abbreviation: NA, not applicable.

^a^ Age available for 195 eligible and declining patients; sex, 198; race, 192; ethnicity, 191; diagnosis, 191; on active treatment, 68.

^b^ Two-sided *P* values were reported from *t* test.

^c^ Two-sided *P* values were reported from Fisher exact test.

^d^ Two-sided *P* values were reported from Pearson χ^2^ test.

^e^ For declining patients, partial data were only collected.

Bayesian estimation of the model reached convergence (PSR <1.10) after 5000 iterations, and the PSR did not bounce over more iterations. The 95% CrIs for the difference between the observed and the model estimated χ^2^ statistics (−46.547 to 64.334) centered around 0, and the posterior predictive *P *value was .37, indicating a good model fit. Selected structural equation modeling results are shown in Table 4. Meaning and peace were significantly inversely associated with anxiety (β = –7.94; 95% CI, –12.88 to –4.12), depression (β = –10.49; 95% CI, –15.92 to –6.50), and fatigue (β = –8.901; 95% CI, –15.34 to –3.61). Several religious and spiritual variables also had significant inverse associations with PROMIS outcomes through associations with meaning/peace. Feeling God’s presence daily and considering oneself very religious were inversely associated with anxiety (feeling God’s presence: β = –3.37; 95% CI, –6.82 to –0.95; very religious: β = –2.81; 95% CI, –6.06 to –0.45) and with depression (feeling God’s presence: β = –4.50; 95% CI, –8.51 to –1.40; very religious: β = –3.77; 95% CI, –7.68 to –0.61). Considering oneself very spiritual was positively associated with anxiety (β = 2.11; 95% CI, 0.05 to 4.95) and depression (β = 2.84; 95% CI, 0.07 to 6.29). Feeling God’s presence daily and considering oneself very religious were associated with fatigue (feeling God’s presence: β = –3.73; 95% CI, –8.03 to –0.90; very religious: β = –3.11; 95% CI, –7.31 to –0.40). Thus, an AYA reporting higher anxiety, depression, or fatigue was highly likely to also report lower levels of meaning/peace and of feeling God’s presence daily. There were no significant findings for pain interference among the PROs or for daily prayer or attending religious services weekly among the religiousness variables.

**Table 4.** Bivariate Associations of Spiritual and Religious Items With Patient-Reported Outcomes for 126 Adolescents and Young Adults

Brief MMRS Variable	PROMIS, mean (SD)			
	Anxiety T-score	Depression T-score	Pain interference T-score	Fatigue T-score
1. I feel God’s presence				
Many times a day or every day (n = 39)	44 (9.7)	42.6 (9.7)	44.5 (11)	45.5 (14)
Most days, some days, once in a while, or never or almost never (n = 83)	48 (9.1)	46.2 (9.8)	43.2 (9.8)	45.3 (11.7)
*P* value	.03	.06	.52	.93
12. How often do you pray privately; that is, how often do you pray in settings other than a church, synagogue, mosque, or other place of worship and at times when you are not attending functions of a religiously based group?				
More than once a day or once a day (n = 37)	45.4 (9.4)	42.9 (8.6)	44.8 (10.8)	44.9 (12.5)
A few times a week, once a week, a few times a month, once a month, less than once a month, or never (n = 87)	47.4 (9.5)	46.1 (10.3)	43.6 (10.2)	45.7 (12.5)
*P* value	.29	.10	.56	.75
34. How often do you go to religious services?				
More than once a week or every week or more often (n = 41)	44.8 (9.1)	44.1 (10.4)	45.7 (11.4)	44.5 (13.9)
Once or twice a month, every month or so, once or twice a year, or never (n = 82)	47.9 (9.5)	45.6 (9.7)	43.2 (9.8)	46.1 (11.8)
*P* value	.09	.42	.21	.50
37. To what extent do you consider yourself a religious person?				
Very religious or moderately religious (n = 72)	46.1 (9.6)	43.6 (9.7)	43.5 (10.4)	44.2 (12.7)
Slightly religious or not religious at all (n = 54)	47.6 (9.2)	46.9 (10)	44.2 (10.4)	47 (12.1)
*P* value	.38	.06	.71	.21
38. To what extent do you consider yourself a spiritual person?				
Very spiritual or moderately spiritual (n = 68)	48.5 (10.4)	46.8 (10.2)	44.2 (10.6)	46.9 (13)
Slightly spiritual or not spiritual at all (n = 58)	44.7 (7.8)	43 (9.3)	43.4 (10)	43.6 (11.7)
*P* value	.02	.03	.67	.14

Abbreviations: MMRS, Multidimensional Measurement of Religiousness/Spirituality; PROMIS, Patient-Reported Outcomes Measurement Information System.

## Discussion

To our knowledge, this is the first study to document an indirect association of meaning and peace with religiousness and spirituality as well as the likelihood of anxiety, depressive symptoms, and fatigue in AYAs with cancer. Specifically, this study went beyond a bivariate approach, demonstrating that feeling God’s presence and identifying as a very religious person were associated with the extent of anxiety, depressive symptoms, and fatigue. The model proposes an indirect association through a sense of meaning and peace. Although the causal direction of these associations cannot be established from our study, these results suggest that a novel and potentially efficacious intervention target may be a sense of meaning and peace when considering ways to improve anxiety, depression, and fatigue among AYAs with cancer.

Findings from the present study are also consistent with previously published theoretical models and empirical data. Park’s work on meaning, including religious and spiritual meaning,^32,47,48^ posits that religious and spiritual beliefs and practices inform constructed meaning, which is related to health outcomes. Meaning-making coping mediates the association of religiosity with psychological adjustment.^33^ Religion and spirituality are not important to all AYAs; Salsman and colleagues^49^ recommend identifying subgroups for whom dimensions of religion and spirituality are important to their health-related quality of life and offering them interventions that include religion and/or spirituality.^49^

The meaning of a cancer diagnosis is an important factor for AYAs and the adults living with them.^47,48,50,51^ Clinical attention to constructing meaning of the cancer experience is an important element in improving outcomes.^52^ Barakat and colleagues^52^ reported that although distress continued because of having had cancer, finding positive meaning contributed to posttraumatic growth in a sample of 150 AYA cancer survivors and their parents. This is also consistent with findings from a metasynthesis by Kim and colleagues of 51 qualitative studies,^53^ which revealed that constructed meaning fosters resilience and inner growth and that the benefits persist well into survivorship. They noted the different meanings of an adolescent’s cancer experience from their parents’ and suggested that care be individualized for patients and for patient-parent dyads to maximize outcomes. Rosenberg and colleagues reported^54,55^ positive outcomes (ie, resilience, cancer-related quality of life, distress) with skills-based intervention for AYAs with cancer that included meaning as a component. Moskowitz and colleagues^56^ reported improved outcomes in positive affect, antidepressant use, and intrusive or avoidant thoughts using an intervention that included constructed meaning.

The current study’s findings support the inclusion of constructed meaning as part of AYA oncology care.^55^ This approach has also been recognized by the government of the Netherlands, which recently adopted a person-centered definition of health, including attention to meaning and meaninglessness.^57^ Furthermore, that country’s health budget provides for care at home by a recognized spiritual caregiver to address issues of illness-related meaning, focusing on persons older than 50 years and palliative care patients (including children) and their families.^58^ Demonstration of the effects of these outcomes is in progress.

We anticipated finding an association between spiritual constructs and PROs and did not find one. It is possible that no such association exists in this population. It may also be because of the way faith was operationalized. The FACIT faith subscale quantifies the degree of comfort and strength faith provides rather than the magnitude of its importance. Although comfort and strength of faith were not associated with the PROs measured, the actual importance of faith may be motivational, prohealthy behaviors that may relate to PROs. The current study also assessed how pain interferes with life and found no relationship with spiritual or religious variables. Wachholtz and colleagues^59^ reviewed the religious and spiritual literature related to pain, noting that the mixed results between religion, spirituality, and pain may be the result of focusing on a single aspect of the multidimensional experience of pain. Pain interference may not be an aspect of pain associated with the religious and spiritual constructs quantified by the measures used in this study. It is also possible that the model used by Wachholtz and colleagues^59^ is not fully applicable for AYAs.

This study has important clinical implications. All pediatricians and adolescent medicine specialists should practice primary palliative care to minimize AYAs’ suffering in any form. Primary palliative care comprises basic evaluation and management of symptoms and facilitated conversations about goals of care and advance care planning.^60^ Although many pediatric providers may be reluctant to address these issues, AYAs want providers to address their concerns, including spiritual concerns, and their desire for these to be addressed increases with their disease acuity.^61,62,63^ Steinhauser and colleagues have demonstrated the efficacy of a 1-question intervention among adults, asking, “Are you at peace?”^64^ Such simple, nonthreatening interventions may be a feasible way to explore the topic of peace with AYAs. Their sense of peace and their expressed needs for dealing with death and dying may provide opportunities for the broader use of interdisciplinary palliative care teams.

Specialty care addressing meaning and peace to improve outcomes may take several forms. Referrals to psychologists, who routinely deal with issues of spirituality, meaning, and health, may be appropriate.^32,51^ Individual and group interventions addressing meaning for people with cancer have shown efficacy for increasing spiritual well-being and for decreasing anxiety, depression, and pain.^65,66,67,68^ Referrals for specialty spiritual care from clinically trained chaplains may also be beneficial.^69^ Chaplains are trained to work with existential questions of meaning within the framework of the patient’s beliefs.^70,71^

Meaningful conclusions can be drawn from this study, moving the state of the science of spirituality forward.^72^ Meaning-making is a complex^73^ but modifiable process. Clinical application of these findings could facilitate further integration of religious considerations and meaning-making into pediatric palliative care,^74^ as has been demonstrated with adults.^75^

### Limitations

This study has several limitations. Cross-sectional data do not permit examination of causality, and longitudinal data were not available. There are no universally accepted definitions of spirituality and religion among researchers.^76,77^ Participants self-defined these terms when completing the questionnaires; the results may be confounded through the use of multiple definitions, although there is evidence that AYAs define these terms similarly to some reseachers.^76,78^ Several factors limit generalizability. First, it is not possible to generalize beyond the participating population, ie, English-speaking individuals aged 14 to 21 years with cancer in the United States. Second, the sample size dictated a parsimonious model that could not include potential confounders or predicting variables. Finally, the religious and spiritual affiliations of participants did not reflect the US demographic characteristics for this age group. There was a risk of participants providing socially desirable responses by having questions read aloud, although responses were entered by a research assistant who was trained to ask the questions in a way that would minimize bias. If adolescents preferred to enter their own responses, they were permitted; this rarely occurred. Furthermore, this was an analysis of baseline data informing an advance care planning trial. Male patients were more likely to decline participation in the primary study; thus, selection bias may affect the generalizability of these results. Nevertheless, strengths of this study include the application of rigorous scientific methods. First, 39% of those approached agreed to participate. While this is lower than participation rates in psychosocial intervention trials and represents a limitation,^79^ it is better than the 20% or lower participation rates of individuals aged 15 to 19 years in clinical trials, an enrollment problem identified by the US Centers for Disease Control and Prevention.^80,81,82^ Second, use of validated and reliable questionnaires increased replicability and transparency. Third, 99.5% of the data were complete. Fourth, the use of structural equation modeling to identify indirect associations between meaning and peace and/or faith on physical and emotional symptoms addresses weaknesses in the rigor of previous research.

## Conclusions

Study results demonstrated how multiple facets of religion and spirituality were associated with anxiety, depression, and fatigue among AYAs with cancer, which were associated with their sense of meaning and peace. Future research based on this study could explore the extent to which it represents a novel and potentially efficacious focus for intervention development to improve quality of life for AYAs after a cancer diagnosis.

## Notes

Supplement. **eFigure.** Initial Model of the Role of Spirituality (Meaning/Peace and Faith) and Religiousness on Patient-Reported Symptoms Among Adolescents and Young Adults With Cancer: Structured Equation Model

## References

[1] Siegel RL, Miller KD, Jemal A Cancer statistics, 2019. CA Cancer J Clin. 2019;69(1):7-34. doi:10.3322/caac.21551 30620402

[2] Foster TL, Bell CJ, Gilmer MJ Symptom management of spiritual suffering in pediatric palliative care. J Hospice Palliative Nurs. 2012;14(2):109-115. doi:10.1097/NJH.0b013e3182491f4b

[3] Wolfe J, Hammel JF, Edwards KE, Easing of suffering in children with cancer at the end of life: is care changing? J Clin Oncol. 2008;26(10):1717-1723. doi:10.1200/JCO.2007.14.0277 18375901

[4] Ullrich CK, Dussel V, Orellana L, Self-reported fatigue in children with advanced cancer: results of the PediQUEST study. Cancer. 2018;124(18):3776-3783. doi:10.1002/cncr.31639 30291811 PMC6214736

[5] Wolfe J, Orellana L, Ullrich C, Symptoms and distress in children with advanced cancer: prospective patient-reported outcomes from the PediQUEST Study. J Clin Oncol. 2015;33(17):1928-1935. doi:10.1200/JCO.2014.59.1222 25918277 PMC4451175

[6] National Consensus Project for Quality Palliative Care Clinical Practice Guidelines for Quality Palliative Care. 4th ed National Coalition for Hospice and Palliative Care; 2018.

[7] Smith GA US public becoming less religious. Published November 3, 2015 Accessed September 2, 2019. https://www.pewforum.org/2015/11/03/u-s-public-becoming-less-religious/

[8] Smith G Religious landscape study. Accessed February 6, 2020. https://www.pewforum.org/religious-landscape-study/religious-tradition/unaffiliated-religious-nones/

[9] Barton KS, Tate T, Lau N, Taliesin KB, Waldman ED, Rosenberg AR “I’m not a spiritual person”: how hope might facilitate conversations about spirituality among teens and young adults with cancer. J Pain Symptom Manage. 2018;55(6):1599-1608. doi:10.1016/j.jpainsymman.2018.02.001 29428188 PMC5951752

[10] Pargament KI, Murray-Swank N, Magyar-Russell GM, Ano G. Spiritual struggle: a phenomenon of interest to psychology and religion In: Miller WR, Delaney HD, eds. Judeo-Christian Perspectives on Psychology: Human Nature, Motivation, and Change. APA Press; 2005:245-268.

[11] Ai AL, Pargament KI, Appel HB, Kronfol Z Depression following open-heart surgery: a path model involving interleukin-6, spiritual struggle, and hope under preoperative distress. J Clin Psychol. 2010;66(10):1057-1075. doi:10.1002/jclp.20716 20593431

[12] Pargament KI, Koenig HG, Tarakeshwar N, Hahn J Religious coping methods as predictors of psychological, physical and spiritual outcomes among medically ill elderly patients: a two-year longitudinal study. J Health Psychol. 2004;9(6):713-730. doi:10.1177/1359105304045366 15367751

[13] Cotton S, Zebracki K, Rosenthal SL, Tsevat J, Drotar D Religion/spirituality and adolescent health outcomes: a review. J Adolesc Health. 2006;38(4):472-480. doi:10.1016/j.jadohealth.2005.10.005 16549317

[14] Desrosiers A, Miller L Relational spirituality and depression in adolescent girls. J Clin Psychol. 2007;63(10):1021-1037. doi:10.1002/jclp.20409 17828762

[15] Harris SK, Sherritt LR, Holder DW, Kulig J, Shrier LA, Knight JR Reliability and validity of the Brief Multidimensional Measure of Religiousness/Spirituality among adolescents. J Relig Health. 2008;47(4):438-457. doi:10.1007/s10943-007-9154-x 19093673

[16] Dew RE, Daniel SS, Armstrong TD, Goldston DB, Triplett MF, Koenig HG Religion/spirituality and adolescent psychiatric symptoms: a review. Child Psychiatry Hum Dev. 2008;39(4):381-398. doi:10.1007/s10578-007-0093-2 18219572

[17] Baetz M, Bowen R Chronic pain and fatigue: associations with religion and spirituality. Pain Res Manag. 2008;13(5):383-388. doi:10.1155/2008/263751 18958309 PMC2799261

[18] Curtin KB, Watson AE, Wang J, Okonkwo OC, Lyon ME Pediatric Advance Care Planning (pACP) for teens with cancer and their families: design of a dyadic, longitudinal RCCT. Contemp Clin Trials. 2017;62:121-129. doi:10.1016/j.cct.2017.08.016 28844985 PMC5641257

[19] Harris PA, Taylor R, Thielke R, Payne J, Gonzalez N, Conde JG Research electronic data capture (REDCap)—a metadata-driven methodology and workflow process for providing translational research informatics support. J Biomed Inform. 2009;42(2):377-381. doi:10.1016/j.jbi.2008.08.010 18929686 PMC2700030

[20] Hinds PS, Nuss SL, Ruccione KS, PROMIS pediatric measures in pediatric oncology: valid and clinically feasible indicators of patient-reported outcomes. Pediatr Blood Cancer. 2013;60(3):402-408. doi:10.1002/pbc.24233 22829446

[21] Menard JC, Hinds PS, Jacobs SS, Feasibility and acceptability of the Patient-Reported Outcomes Measurement Information System measures in children and adolescents in active cancer treatment and survivorship. Cancer Nurs. 2014;37(1):66-74. doi:10.1097/NCC.0b013e3182a0e23d 24036439 PMC3859798

[22] DeWitt EM, Stucky BD, Thissen D, Construction of the eight-item Patient-Reported Outcomes Measurement Information System pediatric physical function scales: built using item response theory. J Clin Epidemiol. 2011;64(7):794-804. doi:10.1016/j.jclinepi.2010.10.012 21292444 PMC3100387

[23] Peterman AH, Fitchett G, Brady MJ, Hernandez L, Cella D Measuring spiritual well-being in people with cancer: the Functional Assessment of Chronic Illness Therapy—Spiritual Well-being Scale (FACIT-Sp). Ann Behav Med. 2002;24(1):49-58. doi:10.1207/S15324796ABM2401_06 12008794

[24] Fetzer/National Institute on Aging WG Multidimensional Measurement of Religiousness/Spirituality for Use in Health Research: A Report of the Fetzer/National Institute on Aging Working Group. Fetzer Institute; 1999.

[25] Masters KS, Carey KB, Maisto SA, Caldwell PE, Wolfe TV, Hackney HL Psychometric examination of the brief multidimensional measure of religiousness/spirituality among college students. Int J Psychol Relig. 2009;19(2):106-120. doi:10.1080/10508610802711194

[26] Weaver MS, Heinze KE, Bell CJ, ; Pediatric Palliative Care Special Interest Group at Children’s National Health System Establishing psychosocial palliative care standards for children and adolescents with cancer and their families: an integrative review. Palliat Med. 2016;30(3):212-223. doi:10.1177/0269216315583446 25921709 PMC4624613

[27] Cotton S, McGrady ME, Rosenthal SL Measurement of religiosity/spirituality in adolescent health outcomes research: trends and recommendations. J Relig Health. 2010;49(4):414-444. doi:10.1007/s10943-010-9324-0 20127172 PMC2917535

[28] Grossoehme DH, Szczesniak RD, Britton LL, Adherence determinants in cystic fibrosis: cluster analysis of parental psychosocial, religious, and/or spiritual factors. Ann Am Thorac Soc. 2015;12(6):838-846. doi:10.1513/AnnalsATS.201408-379OC 25803407 PMC4590021

[29] Lyon ME, Garvie PA, D’Angelo LJ, ; Adolescent Palliative Care Consortium Advance care planning and HIV symptoms in adolescence. Pediatrics. 2018;142(5):e20173869. doi:10.1542/peds.2017-3869 30341154 PMC6317555

[30] Lyon ME, Kimmel AL, Cheng YI, Wang J The role of religiousness/spirituality in health-related quality of life among adolescents with HIV: a latent profile analysis. J Relig Health. 2016;55(5):1688-1699. doi:10.1007/s10943-016-0238-3 27071797 PMC4958602

[31] Lyon ME, D’Angelo IJ, Cheng YI, Dallas RH, Garvie PA, Wang J The influence of religiousness beliefs and practices on health care decision-making among HIV positive adolescents. AIDS Care. Published online September 19, 2019. doi:10.1080/09540121.2019.1668523 PMC7080568 31535560

[32] Park CL Religiousness/spirituality and health: a meaning systems perspective. J Behav Med. 2007;30(4):319-328. doi:10.1007/s10865-007-9111-x 17522971

[33] Park CL Religion as a meaning-making framework in coping with life stress. J Soc Issues. 2005;61(4):707-729. doi:10.1111/j.1540-4560.2005.00428.x

[34] Nelson CJ, Rosenfeld B, Breitbart W, Galietta M Spirituality, religion, and depression in the terminally ill. Psychosomatics. 2002;43(3):213-220. doi:10.1176/appi.psy.43.3.213 12075036

[35] Pirutinsky S, Rosmarin DH, Pargament KI, Midlarsky E Does negative religious coping accompany, precede, or follow depression among Orthodox Jews? J Affect Disord. 2011;132(3):401-405. doi:10.1016/j.jad.2011.03.015 21439650

[36] Muthen L, Muthen B Mplus User’s Guide. Muthen & Muthen; 2017.

[37] Lee SY, Song XY Evaluation of the Bayesian and maximum likelihood approaches in analyzing structural equation models with small sample sizes. Multivariate Behav Res. 2004;39(4):653-686. doi:10.1207/s15327906mbr3904_4 26745462

[38] Muthén B, Asparouhov T Bayesian structural equation modeling: a more flexible representation of substantive theory. Psychol Methods. 2012;17(3):313-335. doi:10.1037/a0026802 22962886

[39] Muthen B Bayesian analysis in Mplus: a brief introduction. Published 2010 Accessed February 12, 2020. http://www.statmodel.com/download/IntroBayesVersion%201.pdf

[40] Enders CK Applied Missing Data Analysis. Guilford Press; 2010.

[41] Enders C, Bandalos D The relative performance of full information maximum likelihood estimation for missing data in structural equation models. Struct Equ Modeling. 2001;8(3):430-457. doi:10.1207/S15328007SEM0803_5

[42] Graham JW Missing data analysis: making it work in the real world. Annu Rev Psychol. 2009;60:549-576. doi:10.1146/annurev.psych.58.110405.085530 18652544

[43] Little RJ, Rubin DB Statistical Analysis With Missing Data. 2nd ed Wiley; 2002. doi:10.1002/9781119013563

[44] Gelman A, Meng X, Stern H Posterior predictive assessment of model fitness via realized discrepancies. Stat Sin. 1996;6(4):733-760. Accessed May 1, 2020. http://www.stat.columbia.edu/~gelman/research/published/A6n41.pdf

[45] Gelman A, Carlin JB, Stern HS, Rubin DB Bayesian Data Analysis. Chapman and Hall/CRC Press; 2004.

[46] Gill J Bayesian Methods: A Social and Behavioral Science Approach. 2nd ed Chapman and Hall; 2008.

[47] Park CL, Edmondson D, Fenster JR, Blank TO Meaning making and psychological adjustment following cancer: the mediating roles of growth, life meaning, and restored just-world beliefs. J Consult Clin Psychol. 2008;76(5):863-875. doi:10.1037/a0013348 18837603

[48] Park CL, Edmondson D, Hale-Smith A, Blank TO Religiousness/spirituality and health behaviors in younger adult cancer survivors: does faith promote a healthier lifestyle? J Behav Med. 2009;32(6):582-591. doi:10.1007/s10865-009-9223-6 19639404

[49] Salsman JM, Yost KJ, West DW, Cella D Spiritual well-being and health-related quality of life in colorectal cancer: a multi-site examination of the role of personal meaning. Support Care Cancer. 2011;19(6):757-764. doi:10.1007/s00520-010-0871-4 20405147 PMC4474154

[50] Alcorn SR, Balboni MJ, Prigerson HG, “If God wanted me yesterday, I wouldn’t be here today”: religious and spiritual themes in patients’ experiences of advanced cancer. J Palliat Med. 2010;13(5):581-588. doi:10.1089/jpm.2009.0343 20408763

[51] Park CL, Cho D Spiritual well-being and spiritual distress predict adjustment in adolescent and young adult cancer survivors. Psychooncology. 2017;26(9):1293-1300. doi:10.1002/pon.4145 27196993

[52] Barakat LP, Alderfer MA, Kazak AE Posttraumatic growth in adolescent survivors of cancer and their mothers and fathers. J Pediatr Psychol. 2006;31(4):413-419. doi:10.1093/jpepsy/jsj058 16093518

[53] Kim B, White K, Patterson P Understanding the experiences of adolescents and young adults with cancer: a meta-synthesis. Eur J Oncol Nurs. 2016;24:39-53. doi:10.1016/j.ejon.2016.06.002 27697276

[54] Rosenberg AR, Bradford MC, McCauley E, Promoting resilience in adolescents and young adults with cancer: results from the PRISM randomized controlled trial. Cancer. 2018;124(19):3909-3917. doi:10.1002/cncr.31666 30230531

[55] Rosenberg AR, Yi-Frazier JP, Wharton C, Gordon K, Jones B Contributors and inhibitors of resilience among adolescents and young adults with cancer. J Adolesc Young Adult Oncol. 2014;3(4):185-193. doi:10.1089/jayao.2014.0033 25969794 PMC4418509

[56] Moskowitz JT, Carrico AW, Duncan LG, Randomized controlled trial of a positive affect intervention for people newly diagnosed with HIV. J Consult Clin Psychol. 2017;85(5):409-423. doi:10.1037/ccp0000188 28333512 PMC5398944

[57] Huber M, van Vliet M, Giezenberg M, Towards a ‘patient-centred’ operationalisation of the new dynamic concept of health: a mixed methods study. BMJ Open. 2016;6(1):e010091. doi:10.1136/bmjopen-2015-010091 26758267 PMC4716212

[58] van Twillert M Toch 10 miljoen erbij voor geestelijk verzorger eerste lijn. *Medisch Contact* Published January 14, 2019 Accessed October 15, 2019. https://www.medischcontact.nl/nieuws/laatste-nieuws/artikel/toch-10-miljoen-erbij-voor-geestelijk-verzorger-eerste-lijn.htm

[59] Wachholtz AB, Fitch CE, Makowski S, Tjia J A comprehensive approach to the patient at end of life: assessment of multidimensional suffering. South Med J. 2016;109(4):200-206. doi:10.14423/SMJ.0000000000000439 27043799 PMC4824542

[60] Friedrichsdorf SJ, Remke S, Hauser J, Development of a pediatric palliative care curriculum and dissemination model: Education in Palliative and End-of-Life Care (EPEC) pediatrics. J Pain Symptom Manage. 2019;58(4):707-720.e3. doi:10.1016/j.jpainsymman.2019.06.008 31220594 PMC6754756

[61] Grossoehme DH, Ragsdale JR, McHenry CL, Thurston C, DeWitt T, VandeCreek L Pediatrician characteristics associated with attention to spirituality and religion in clinical practice. Pediatrics. 2007;119(1):e117-e123. doi:10.1542/peds.2006-0642 17200236

[62] Feudtner C, Haney J, Dimmers MA Spiritual care needs of hospitalized children and their families: a national survey of pastoral care providers’ perceptions. Pediatrics. 2003;111(1):e67-e72. doi:10.1542/peds.111.1.e67 12509597

[63] King SD, Dimmers MA, Langer S, Murphy PE Doctors’ attentiveness to the spirituality/religion of their patients in pediatric and oncology settings in the northwest USA. J Health Care Chaplain. 2013;19(4):140-164. doi:10.1080/08854726.2013.829692 24070435

[64] Steinhauser KE, Voils CI, Clipp EC, Bosworth HB, Christakis NA, Tulsky JA “Are you at peace?”: one item to probe spiritual concerns at the end of life. Arch Intern Med. 2006;166(1):101-105. doi:10.1001/archinte.166.1.101 16401817

[65] Cole B, Pargament K Re-creating your life: a spiritual/psychotherapeutic intervention for people diagnosed with cancer. Psychooncology. 1999;8(5):395-407. doi:10.1002/(SICI)1099-1611(199909/10)8:5<395::AID-PON408>3.0.CO;2-B 10559799

[66] Cole B Spiritually-focused psychotherapy for people diagnosed with cancer: a pilot outcomes study. Ment Health Relig Cult. 2005;8(3):217-226. doi:10.1080/13694670500138916

[67] Breitbart W, Poppito S, Rosenfeld B, Pilot randomized controlled trial of individual meaning-centered psychotherapy for patients with advanced cancer. J Clin Oncol. 2012;30(12):1304-1309. doi:10.1200/JCO.2011.36.2517 22370330 PMC3646315

[68] Breitbart W, Rosenfeld B, Pessin H, Applebaum A, Kulikowski J, Lichtenthal WG Meaning-centered group psychotherapy: an effective intervention for improving psychological well-being in patients with advanced cancer. J Clin Oncol. 2015;33(7):749-754. doi:10.1200/JCO.2014.57.2198 25646186 PMC4334778

[69] Macdonald GW Primary care chaplaincy: an intervention for complex presentation. Prim Health Care Res Dev. 2018:1-12. 30295211 10.1017/S1463423618000737 PMC8512567

[70] Cadge W, Sigalow E Negotiating religious differences: the strategies of interfaith chaplains in healthcare. J Sci Study Relig. 2013;52(1):146-158. doi:10.1111/jssr.12008

[71] Strang S, Strang P Questions posed to hospital chaplains by palliative care patients. J Palliat Med. 2002;5(6):857-864. doi:10.1089/10966210260499041 12685532

[72] Balboni TA, Fitchett G, Handzo GF, State of the science of spirituality and palliative care research, part II: screening, assessment, and interventions. J Pain Symptom Manage. 2017;54(3):441-453. doi:10.1016/j.jpainsymman.2017.07.029 28734881

[73] Park CL Making sense of the meaning literature: an integrative review of meaning making and its effects on adjustment to stressful life events. Psychol Bull. 2010;136(2):257-301. doi:10.1037/a0018301 20192563

[74] Wiener L, McConnell DG, Latella L, Ludi E Cultural and religious considerations in pediatric palliative care. Palliat Support Care. 2013;11(1):47-67. doi:10.1017/S1478951511001027 22617619 PMC3437238

[75] Otis-Green S, Ferrell B, Borneman T, Puchalski C, Uman G, Garcia A Integrating spiritual care within palliative care: an overview of nine demonstration projects. J Palliat Med. 2012;15(2):154-162. doi:10.1089/jpm.2011.0211 22304654

[76] Hill PC, Pargament KI, Hood RW, Conceptualizing religion and spirituality: points of commonality, points of departure. J Theory Soc Behav. 2000;30(1):51-77. doi:10.1111/1468-5914.00119

[77] Zinnbauer BJ, Pargament KI, Cole B, Religion and spirituality: unfuzzying the fuzzy. J Sci Study Relig. 2000;36(4):549-564. doi:10.2307/1387689

[78] Raftopoulos M, Bates G ‘It’s that knowing that you are not alone’: the role of spirituality in adolescent resilience. Int J Child Spiritual. 2011;16(2):151-167. doi:10.1080/1364436X.2011.580729

[79] Rosenberg AR, Junkins CC, Sherr N, Conducting psychosocial intervention research among adolescents and young adults with cancer: lessons from the PRISM randomized clinical trial. Children (Basel). 2019;6(11):E117. doi:10.3390/children6110117 31652895 PMC6915330

[80] Tai E, Beaupin L, Bleyer A Clinical trial enrollment among adolescents with cancer: supplement overview. Pediatrics. 2014;133(suppl 3):S85-S90. doi:10.1542/peds.2014-0122B 24918212 PMC6069529

[81] Ferrari A, Bleyer A Participation of adolescents with cancer in clinical trials. Cancer Treat Rev. 2007;33(7):603-608. doi:10.1016/j.ctrv.2006.11.005 17250970

[82] Bleyer WA Cancer in older adolescents and young adults: epidemiology, diagnosis, treatment, survival, and importance of clinical trials. Med Pediatr Oncol. 2002;38(1):1-10. doi:10.1002/mpo.1257 11835231

