# Supplementary material for: Association of Religious and Spiritual Factors With Patient-Reported Outcomes of Anxiety, Depressive Symptoms, Fatigue, and Pain Interference Among Adolescents and Young Adults With Cancer
Source: JAMA Netw Open. 2020 Jun 16;3(6):e206696. doi: 10.1001/jamanetworkopen.2020.6696 (PMC7298609; doi:10.1001/jamanetworkopen.2020.6696)
Supplement: Supplement. — eFigure. Initial Model of the Role of Spirituality (Meaning/Peace and Faith) and Religiousness on Patient-Reported Symptoms Among Adolescents and Young Adults With Cancer: Structured Equation Model [file jamanetwopen-3-e206696-s001.pdf]

Supplementary Online Content

Grossoehme DH, Friebert S, Baker JN, et al. Association of religious and spiritual factors with patient-reported outcomes of anxiety, depressive symptoms, fatigue, and pain interference among adolescents and young adults with cancer. *JAMA Netw Open*. 2020;3(6):e206696. doi:10.1001/jamanetworkopen.2020.6696

eFigure. Initial Model of the Role of Spirituality (Meaning/Peace and Faith) and Religiousness on Patient-Reported Symptoms Among Adolescents and Young Adults With Cancer: Structured Equation Model

This supplementary material has been provided by the authors to give readers additional information about their work.

© 2020 Grossoehme DH et al. *JAMA Network Open*.

eFigure. Initial Model of the Role of Spirituality (Meaning/Peace and Faith) and Religiousness on Patient-Reported Symptoms Among Adolescents and Young Adults With Cancer: Structured Equation Model

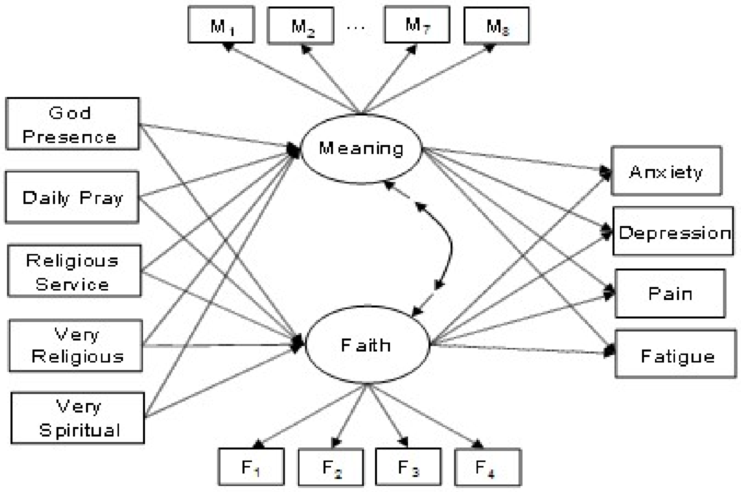

M Meaning/peace items
F: Faith items

© 2020 Grossoehme DH et al. *JAMA Network Open*.
